# 

**Published:** 1878-06

**Authors:** 


### ARTICLE VII.

### *California State Dental Association.*

The Ninth Annual Session of the California State Dental Association will be held in San Francisco, June 4th, and continue four days.

Previous sessions have been of great benefit to the profession, and we confidently hope the coming one will equal, if not excel, in interest and usefulness, any that have preceded it.

It is expected that more time will be given to Clinical operations than has been during former sessions, for by this means it is believed more may be accomplished than by any other.

The Elevation of the Profession is the first object we have in view, and it is earnestly desired that each member will feel that he should contribute to the common fund of nowledge, from which we may all freely draw.

That the coming session may be made one of unusual interest, you are respectfully urged to suspend Dental Operations in your office for a few days, and unite in an endeavor to make the Ninth Annual Session one of pleasure and profit to all.

WM. B. KINGSBURY,
*Pres. California State Dental Association.*

---

### *South Carolina State Dental Association.*

The Eighth Annual Meeting of the South Carolina State Dental Association, J. R. Thompson, D. D. S., President, will be held in the city of Columbia on Tuesday, June 4th, 1878, at Half-Past 8 o'clock P. M.

There will be Essays, Discussions and Clinics ; also a large assortment of Dental Material and Instruments, including the latest improvements, as per Resolution of last meeting.

The various Railroads are requested to pass Delegates at reduced rates.

The State Board of Dental Examiners will grant License to candidates who pass a satisfactory examination.

*Executive Committee.*—Dr. W. L. Reynolds, Dr. T. T. Moore, Dr. D. L. Boozer.

G. F. S. WRIGHT, *Rec. Secretary.*

---

### *American Dental Association.*

The eighteenth annual Session of the American Dental Association will be held at Niagara, Tuesday, August 6th, 1878, and continue in Session four days.

MARSHALL H. WEBB, *Cor. Secretary.*

### *Southern Dental Association.*

The Tenth Annual Meeting of the Southern Dental Association will be held at Niagara Falls on Tuesday, August 6th, 1878.

The change of place of meeting, from Atlanta, Ga., to Niagara Falls, has been deemed advisable for the purpose of joining the American Dental Association and the American Dental Convention, which meet at the same time and place; when an effort will be made to organize an American Dental Congress, or some sort of a truly national organization.

Committees of Conference have been appointed from the three societies for this purpose. These facts will enhance the interest, and probably secure a large attendance. An unusually interesting meeting may be expected and the profession is invited.

S. J. Cobb, *President*,

Nashville, Tenn.

E. S. Chisholm, *Sec'y*

Tuscaloosa, Ala.

---

### EDITORIAL, ETC.

### To Readers and Correspondents.

Communications intended for publication, and exchange journals and papers, should be addressed to the Editor of the American Journal of Dental Science, 259 N. Eutaw St., Baltimore, Md. All letters relating to the business of the journal, or containing cash remittances, to be directed to Messrs. Snowden & Cowman. Articles for publication should be received by the editor at least three weeks previous to the first day of the month on which the number in which it is designed for them to appear, is issued.

### MONTHLY SUMMARY.

☞ The American Journal of Dental Science will contain fifty pages of reading matter in each number, making a volume of about

*Six Hundred* pages

EXCLUSIVE OF ADVERTISEMENTS

THE

### AMERICAN JOURNAL

OF

### DENTAL SCIENCE.

The *Journal* is issued on the first of each month and contains not less than *fifty* pages of reading matter in each number, exclusive of advertisements, making a volume of *six hundred* pages per annum.

In each number will be found Original Communications, Correspondence, Selected Articles, Monthly Summary, Bibliographical Notices and Editorial Matter, and every effort will be exerted to render the *Journal* worthy of the patronage of the Dental profession.

A generous co-operation is respectfully solicited from the members of the profession; and as the *Journal* has now a printing office of its own, which materially lessens the cost of its publication, it will be issued at the reduced subscription price of

**$2.50 Per Annum in Advance.**

Communications are respectfully solicited on all subjects pertaining to the practice of dentistry, as well as reports of cases occurring in dental practice.

### RATES OF ADVERTISING.

| | 1 MO. | 2 MO. | 3 MO. | 6 MO. | 12 MO. |
|---|---|---|---|---|---|
| 1 PAGE. | $15 00 | $22 50 | $30 00 | $50 00 | $100 00 |
| ½ " | 10 00 | 15 00 | 20 00 | 30 00 | 50 00 |
| ¼ " | 7 00 | 11 00 | 15 00 | 20 00 | 30 00 |
| ⅛ " | 5 00 | 8 00 | 11 00 | 15 00 | 20 00 |

All original communications designed for publication, works for review, new instruments for notice, exchanges, &c., should be directed to the editor, 259 N. Eutaw St., Baltimore, Md.

All advertisements and subscriptions should be addressed to

SNOWDEN & COWMAN,
*Publishers of the Am. Journal of Dental Science.*
86 W. FAYETTE ST. BET. CHARLES & LIBERTY STS.,
BALTIMORE, MD

